# Variation in performance on common content items at UK medical schools

**DOI:** 10.1186/s12909-021-02761-1

**Published:** 2021-06-05

**Authors:** David Hope, David Kluth, Matthew Homer, Avril Dewar, Richard Fuller, Helen Cameron

**Affiliations:** 1 Medical Education Unit, Edinburgh Medical School, The Chancellor’s Building, College of Medicine and Veterinary Medicine, The University of Edinburgh, 49 Little France Crescent, EH16 4SB Edinburgh, United Kingdom; 2 Leeds School of Medicine, Worsley Building, Leeds Institute of Medical Education, University of Leeds, LS2 9JT Leeds, UK; 3 School of Medicine, University of Liverpool, University of Liverpool, Cedar House, Ashton St, L69 3GE Liverpool, UK; 4 Aston Medical School, Aston University, 295 Aston Express Way, B4 7ET Birmingham, UK

## Abstract

**Background:**

Due to differing assessment systems across UK medical schools, making meaningful cross-school comparisons on undergraduate students’ performance in knowledge tests is difficult. Ahead of the introduction of a national licensing assessment in the UK, we evaluate schools’ performances on a shared pool of “common content” knowledge test items to compare candidates at different schools and evaluate whether they would pass under different standard setting regimes. Such information can then help develop a cross-school consensus on standard setting shared content.

**Methods:**

We undertook a cross-sectional study in the academic sessions 2016-17 and 2017-18. Sixty “best of five” multiple choice ‘common content’ items were delivered each year, with five used in both years. In 2016-17 30 (of 31 eligible) medical schools undertook a mean of 52.6 items with 7,177 participants. In 2017-18 the same 30 medical schools undertook a mean of 52.8 items with 7,165 participants, creating a full sample of 14,342 medical students sitting common content prior to graduation. Using mean scores, we compared performance across items and carried out a “like-for-like” comparison of schools who used the same set of items then modelled the impact of different passing standards on these schools.

**Results:**

Schools varied substantially on candidate total score. Schools differed in their performance with large (Cohen’s *d* around 1) effects. A passing standard that would see 5 % of candidates at high scoring schools fail left low-scoring schools with fail rates of up to 40 %, whereas a passing standard that would see 5 % of candidates at low scoring schools fail would see virtually no candidates from high scoring schools fail.

**Conclusions:**

Candidates at different schools exhibited significant differences in scores in two separate sittings. Performance varied by enough that standards that produce realistic fail rates in one medical school may produce substantially different pass rates in other medical schools – despite identical content and the candidates being governed by the same regulator. Regardless of which hypothetical standards are “correct” as judged by experts, large institutional differences in pass rates must be explored and understood by medical educators before shared standards are applied. The study results can assist cross-school groups in developing a consensus on standard setting future licensing assessment.

## Introduction

Assessment in medical education should ensure doctors are competent, safe practitioners [1, 2]. Typically, candidates approaching registration must sit an “exit” assessment to confirm suitability to work as a doctor [3]. The defensibility of such assessments is of great importance in maintaining the quality of medical education and ensuring patient safety.

Evaluating such assessments can be difficult. In almost all regulatory environments doctors graduate from different institutions. Therefore, a range of institutional contexts, curricula, admissions policies, and resources produce doctors who are nominally equivalent, but differ in experiences [4]. Regulators seek to ensure equivalence across institutions by monitoring and enforcing a shared set of values and requirements [5].

As the content, structure, and weighting of exit assessments vary, direct comparisons across institutions are very difficult to carry out. Several partial solutions have been tested. One approach is to compare candidates on later – usually postgraduate – assessment which can act as a comparative measure. Research has shown that graduates of different medical schools exhibit large differences in performance on postgraduate assessments [6]. Relatedly, evidence has suggested that the performance of individual medical students and doctors exhibits at least moderate stability over time [7, 8] which suggests the variety of candidates applying to medical schools, or their experiences at medical schools, may create meaningful differences between cohorts upon graduation. Performance on (postgraduate) assessment predicts not just technical skill, but professionalism, including the likelihood of being sanctioned while working as a doctor [9, 10]. However, only a small proportion of doctors experience formal sanctioning, and written assessment is only one part of the wider process of professional evaluation.

Collectively, the research on postgraduate performance necessarily contains limitations. Postgraduate attainment can only measure capabilities some years after doctors begin work and cannot confidently identify the source of such differences. Postgraduate assessments are often specialised and sat by only a subset of doctors, and candidates who exit the profession soon after graduation will never sit them.

An alternative method lies in the use of “common content.” Here, a group of institutions pool resources and share assessment content across institutions. So, a group of medical schools may share stations in a clinical examination, or multiple-choice questions (MCQs) in a written examination, with the remaining content set locally and independently. By evaluating both the approach to standard setting and the attainment of different cohorts, it is possible to get a better sense of how variable institutions are, within a single regulatory framework. Research on common content has suggested that different medical schools set very different standards for identical content. Research on MCQ-type written assessment has shown significant differences in medical school standard setting with typically medium effects, with the attendant risk that candidates who passed at institutions with lenient standards would have failed – and potentially not graduated – at institutions with more stringent standards for the same content [11]. A follow-up exploration of standard setting at some of the same schools described institutional, individual, and group factors combining to create highly unique standard setting procedures despite using the same content at all institutions [12].

Research on “common content” clinical examination stations have found similar problems, with standards for the same station varying by up to 13 % between the most lenient and stringent school [3]. Evidence on attainment, rather than standards, remains very sparse but some research on clinical examinations showed medical school cohorts scoring significantly differently on common content stations, in a pool of four medical schools [13].

This is extremely important as it suggests that, even if the content tested in different medical schools is equivalent, the local variability of standards may lead to candidates passing in some environments when they would have failed in others. Indeed, research has suggested that across many measures – content, type, duration, and standard setting – medical schools have a widely varying range of approaches [6, 14]. The fear that monitoring systems do not ensure comparability across institutions has led to recommendations for a knowledge test “licensing” assessment which acts as a single point of measurement for all candidates, alongside a locally designed and delivered clinical performance test, all within a complex regulatory framework [15]. The utility of this proposal remains contested. To some it represents the advance of a test-centric culture where learning is devalued [16] and educational diversity reduced [17]. To others, there are potentially significant benefits to patient safety by harmonising standards [15, 18]. This is an especially challenging area as stakeholders may prioritise different issues: while medical educators may see significant value in a broad range of experiences and curriculum designs, patients and regulators may prioritise high confidence in minimum safety standards. Such licensing assessments may identify genuine differences in attainment between schools, with implications for local standard setting, safety monitoring, and passing rates. Stakeholders might regard a licensing assessment as especially desirable if differences in passing rates are considered to reflect genuine differences in competence. Alternatively as, new doctors currently appear to integrate well into the workplace when adequately supported, [19, 20] a licensing assessment could be an expensive and unnecessary addition.

The practical and theoretical challenges of implementing any multi-site assessment are significant. In the Netherlands, a progress test delivered across institutions has led to a more effective use of resources and enabled cross-school research, but also disagreements over item quality and logistical difficulties in organising the new assessment [21, 22]. In the United States, students have responded to the United States Medical Licensing Examination (USMLE) Step 1 with a range of effective self-directed learning behaviours to maximise the likelihood of passing [23]. However, the focus on the candidate’s USMLE score has led authors to claim other aspects of performance – including achievements during medical school – have been under-valued, which has in turn led to reporting changes whereby only the candidate’s pass/fail status is reported [24]. Such research demonstrates that cross-school assessment inevitably has serious implications for curriculum design and student learning even in areas which the assessment does not directly assess.

Despite the potentially significant impacts of a new licensing assessment on passing rates at medical schools, little is known about how such assessment might influence standard setting and pass rates. As a first step, medical educators at all affected schools should be aware of the relative performance of their students and the potential impact of different standard setting regimes, which can in turn help develop a consensus on how to standard set national licensing assessment in a way that recognises educational diversity while also ensuring patient safety.

To develop better evidence in this area, we used “common content” MCQs developed by the Medical Schools Council Assessment Alliance (MSCAA[11]) to compare candidates at 30 medical schools, evaluate performance differences across common content, and estimate the impact of different standards on pass rates ahead of the implementation of a licensing assessment in the United Kingdom.

## Methods

### Context and study design

In the United Kingdom, medical schools are regulated by the General Medical Council (GMC). The GMC sets the standards for undergraduate medical education and defines a series of high-level outcomes which medical schools must meet. [5] UK medical school programmes are typically five or six years long and begin with an introduction to the fundamentals of medicine, anatomy, healthcare in society, and collaborative working. In later years, students rotate through clinical attachments in which they develop knowledge and practical skills in a clinical environment. Before graduation, they sit both written and practical (i.e., clinical) assessment, which must be completed to a satisfactory level before graduating as a doctor. The quantity of assessment at UK medical school varies, with the amount of assessment (in minutes) differing for written (*M* = 2,000, *SD* = 600) and practical assessment (*M* = 500, *SD* = 200). [4] All medical schools in this sample set a locally developed written and clinical examination as part of their final assessment.

We undertook a cross-sectional study in academic sessions 2016-17 and 2017-18. The MSCAA organised 60 core items for participating schools in 2016-17 and 60 in 2017-18, with five used in both years. These were all “single best answer” multiple choice questions with one correct option and four distractors.

The items were curated by the MSCAA Final Clinical Review Group, which is made up of clinicians with expertise in both medical education and the relevant topic areas. Membership in the group rotates so that, over time, all UK medical schools contribute to this group. The common content items were designed to represent the full range of content areas (specialties and learning outcomes) regarded as “core” for a new UK graduate. Items were blueprinted against both GMC Outcomes for Graduates (e.g. “immediate care in medical emergencies”) and content areas (e.g. “respiratory”). So, a question might be blueprinted to test a candidate’s knowledge of respiratory medicine when providing immediate care in a medical emergency. In total 23 content areas were used with an average of 2.6 items per area.

Medical school representatives with expertise in standard setting were invited to comment on the suitability of items and the final set of common content items was designed to be maximally relevant for new doctors. Schools were not obliged to use all items. Items were embedded in each school’s final written assessment [11, 12].

### Participants

All UK medical schools were offered the opportunity to participate in the common content project. All items were delivered within an exit examination sat near the end of medical school. In 2016-17 30 medical schools undertook a mean of 52.6 common content items, with a total candidate number of 7,177. In 2017-18 30 medical schools undertook a mean of 52.8 common content items, with a total candidate number of 7,165, making for 14,342 sittings evaluated within this study. Full details can be found in Table 1. Medical schools had complete control over how many items to use and could use any combination of items.

**Table 1:**
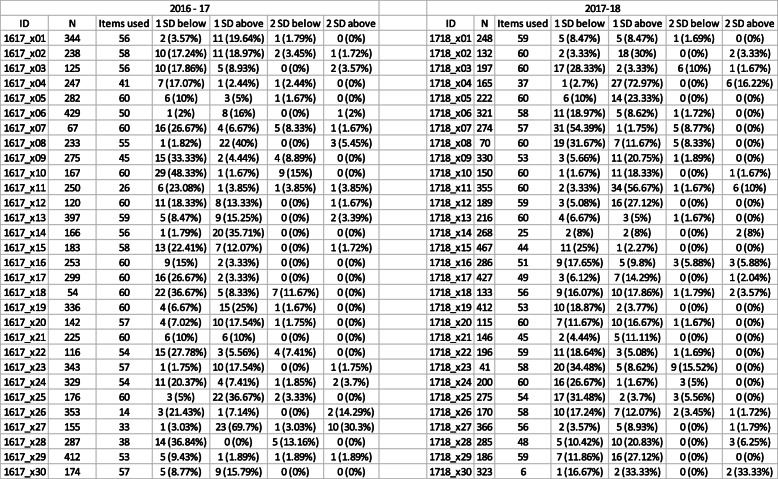
School performance.

Note: School codes were different each year. “SD” = Standard Deviation

### Ethics

 Ethical approval was granted by the University of Edinburgh Medicine and Veterinary Medicine ethics committee. All participant details – both schools and candidates – were anonymised, and the research team had no access to deanonymized data.

### Data collection

Following the completion of assessment, each school reported on the common content items to the MSCAA. This included notes from staff or candidates expressing concerns over item quality and a report of performance per candidate per question. The MSCAA then evaluated the psychometrics of the assessment using a combination of Classical Test Theory (CTT) and Rasch analysis to test whether items were of acceptable quality for analysis. Where a candidate failed to answer a question, this was coded as 0 (incorrect). An exploration of missing responses identified no pattern that would call into question the defensibility of any items or candidate response patterns.

### Data analysis

As medical schools varied in the common content items they selected, making like-for-like comparisons was challenging. We utilised a two-part approach. In part 1, we compared means/facility scores, standard deviations, and discrimination indices for every item for every medical school that used the item. This allowed us to compare the homogeneity of medical schools in terms of both their average score and their variability. We sought to identify where (and how frequently) a given medical school significantly varied compared to other schools to see whether variability could be explained by small deviations across many items, or large deviations in a small number of items. This analysis was intended to be primarily descriptive, though we carried out a formal test of significance (via t-tests) for completeness.

In part 2, we selected a subsample of schools who had all sat a large proportion of the items. 13 schools sat the same 41 items in 2016-17, and 14 schools sat the same 48 items in 2017-18. Note that these numbers refer to the amount of overlapping, shared content. Some schools used more items, but as these were not shared by the entire subsample they were not included in this analysis.

To investigate whether this subset of items differed from the full, 60-item pool, we compared the blueprints of the used and unused item pools. We were unable to identify any pattern of differences in either outcomes or content areas. For example, items on “respiratory” were represented in both pools. These items might differ on the combination of outcomes and content areas, or the specific aspects of respiratory knowledge being investigated, but the high-level outcomes and content areas were equally represented. This most likely represents the decision by the schools included in this subset to select a large, representative sample of items which covered most of the curriculum.

For these schools we carried out a like-for-like analysis of their within-year performance, tested whether performance of the top and bottom tertiles (representing “high scoring” and “low scoring” schools) differed significantly and modelled the impact of different passing standards. An a-priori power calculation showed that analyses used were able to detect small effect sizes at 80 % power [25]. School codes were not re-used, so the same code referred to a different school in each year.

### Part 1 – item performance

We report here a Classical Test Theory (CTT) analysis of the data. While there are advantages to alternative methods – especially Rasch analysis [26] – the comparative simplicity and familiarity of CTT methods were considered desirable given the objective of maximising accessibility for the largest possible audience [27]. While we analysed the data using both a CTT and Rasch framework, only the CTT values are reported here.

For each item, we calculated the overall mean (or facility) score (between zero, indicating no candidate answered the item correctly, and one, indicating all candidates answered correctly), the Standard Deviation (SD) and the discrimination index (a measure of whether the item could discriminate between candidates who performed well or poorly on the assessment as a whole [28]). Facility and discrimination values did not differ significantly between the two study years, indicating the common content operated similarly in each year, and so we repeated the same analysis on each cohort. We calculated mean item facility (*M* = 0.74, *SD* = 0.18) and mean item discrimination on items (*M* = 0.20, *SD* = 0.10). We then calculated mean item performance (and associated SDs) for each school, per year. We then identified the proportion of items where the school was one or two SDs above the mean score, and one or two SDs below the mean score as a measure of the school’s overall performance against all medical schools.

To further explore this, we compared the total number of items where the school scored two SDs below the mean. For the analysis, we compared the bottom and top tertiles and ran the analysis for each cohort. This gave a percentage measurement from zero (the school had no items 2 SDs below the mean) to 100 % (the school’s cohort scored 2 SDs below the mean for every item). We calculated tertiles by the school’s mean mark across all the items they used, and so compared the bottom tertile (the ten lowest performing medical schools on this assessment) against the top tertile (the ten highest performing medical schools).

The main goal of this was not to provide a precise comparison – because schools did not sit exactly the same items this was not possible – but to explore whether differences between schools could be explained by some schools exhibiting much higher rates of incorrect responses across a range of domains. Additionally, this relatively straightforward analysis can be reproduced by medical schools for internal evaluation and to address student queries, without requiring advanced statistical knowledge or significant researcher time. We chose to use 2 SDs as a cutoff as this generally indicated a notably lower score compared to the average school. The observed variance may then reflect differences in teaching approaches and curricula between medical schools, or genuine differences in student competence.

### Part 2 – modelling standards

By comparing item usage across all schools, we identified schools which shared many items. We modelled the interaction of school numbers vs. item numbers: at one extreme it would be possible to compare all schools on a very small number of items, and at the other extreme a very small number of schools on all items. After modelling options, we were able to identify 13 schools from the 2016-17 cohort that had used the same 41 items, and a further 14 schools from the 2017-18 cohort that had used the same 48 items.

This gave us two samples of medical schools sitting identical content. For both years, Cronbach’s alpha = 0.7, indicating an acceptable level of internal consistency for the two sets of items. We compared the bottom and top third of medical schools (rounded for uneven group sizes) in each sample on mean score. As in part 1, the sample size was adequate to test for small effects at 80 % power.

We then modelled the effect of different passing standards. We identified the pass score that would give a score as close as possible to a 5 % fail rate at (a) the four highest-scoring schools (“stringent”) and (b) the four lowest scoring schools (“lenient”). This number was chosen to match the typical fail rate of the Prescribing Safety Assessment (PSA), an assessment sat by candidates across UK medical schools with similar features to future potential licensing assessments [29]. We then estimated the impact of imposing these passing standards on the medical schools. Medical schools received a copy of the results and were able to identify their own school (but not other schools).

## Results

### Part 1 – item performance

In 2016-17, schools in the lowest tertile (that is, their total score on the common items placed them in the lowest third when ranked by performance) had a number of items with facility scores two SD below the mean (*M* = 7.81 %, *SD* = 4.4 %) whereas the top tertile (upper third) had none, a significant difference (t(9) = 5.61, *p* = .001) with a large effect size (*d* = 2.51). This pattern was repeated in 2017-18 with the bottom tertile having some (*M* = 6.62 %, *SD* = 4.19 %) and the top tertile again having none, a significant difference (t(9) = 5, *p* = .001) with a large effect size (*d* = 2.23). This meant that for both years, schools in the bottom tertile reported significantly higher rates of items with facility scores two SD below the mean, indicating a different level of knowledge among those medical school students compared to the top tertile cohorts. This suggests that differences in scores may reflect differences in knowledge across a range of areas.

 A full summary of the medical schools, the number of items they used, their scores relative to other medical schools, and their local sample size can be found in Table 1.

### Part 2 – modelling standards

In 2016-17, comparing the bottom (*M* = 0.76, *SD* = 0.1) and top (*M* = 0.85 *SD* = 0.08) tertiles identified a statistically significant difference (*t*(1570.1) = -20.82, *p* = .001) with a large effect size (*d* = 1.01). This pattern was repeated in 2017-18 where comparing the bottom (*M* = 0.68, *SD* = 0.1) and top (*M* = 0.78, *SD* = 0.09) tertiles identified a statistically significant difference (*t*(1562.5) = − 20.5, *p* = .001), again with a large effect size (*d* = 1.02).

The passing standards diverged with important practical consequences. In 2016-17, the stringent standard was 29.5 (71.95 %) and the lenient standard 24.5 (59.76 %), out of a total of 41. In 2017-18 the stringent standard was 29.73 (61.94 %) and the lenient standard 24 (50 %), out of a total of 48. Table 2 summarises the impact of these illustrative standards on pass rates: applying the most stringent standards to the lowest-scoring medical school would lead to a fail rate of 39.52 % in 2016-17 and 31.98 % in 2017-18. Conversely, applying the lenient standard would lead to one medical school in 2016-17 and four in 2017-18 having no failing candidates at all.

**Table 2:** Like-for-like comparison across schools.

2016–17							2017-18						
School	Mean score	SD score	Tertile	95 % pass score	Stringent % failing	Lenient % failing	School	Mean score	SD score	Tertile	95 % pass score	Stringent % failing	Lenient % failing
x08	0.87	0.07	3	30	2.58 %	0 %	x11	0.82	0.08	3	32	0.85 %	0 %
x19	0.85	0.07	3	30	2.98 %	0.30 %	x12	0.76	0.09	3	30	4.76 %	0 %
x25	0.84	0.08	3	29	6.25 %	0.57 %	x10	0.76	0.09	3	29	5.33 %	0 %
x12	0.82	0.09	3	27	14.17 %	1.67 %	x05	0.75	0.1	3	28	9.91 %	0.90 %
x05	0.81	0.09	2	28	12.77 %	1.42 %	x02	0.75	0.09	2	29	6.82 %	0 %
x15	0.81	0.09	2	26	15.30 %	0.55 %	x29	0.75	0.09	2	28	8.60 %	0.54 %
x21	0.8	0.08	2	27	15.56 %	2.67 %	x28	0.72	0.1	2	27	15.09 %	0.35 %
x16	0.8	0.09	2	26	18.58 %	2.77 %	x13	0.72	0.1	2	27	17.13 %	1.85 %
x04	0.78	0.09	1	25	25.91 %	4.45 %	x01	0.72	0.1	2	26	15.32 %	0.40 %
x07	0.77	0.09	1	25	26.87 %	2.99 %	x20	0.72	0.09	1	28	13.04 %	1.74 %
x18	0.76	0.07	1	26	22.22 %	1.85 %	x22	0.68	0.11	1	25	27.04 %	2.55 %
x17	0.76	0.09	1	24	32.44 %	5.35 %	x24	0.67	0.11	1	23	24.50 %	7.50 %
x10	0.74	0.11	1	22	39.52 %	10.78 %	x08	0.67	0.09	1	25	30.00 %	2.86 %
							x03	0.67	0.11	1	23	31.98 %	5.58 %

Note: school codes were different each year. The “stringent” standard set the pass score as close as possible to yield a 5 % fail rate for the highest scoring medical schools. The “lenient” standard set the pass score as close as possible to yield a 5 % fail rate for the lowest scoring medical schools. For the columns “stringent” and “lenient” the values refer to the percentage of candidates at the medical school who have failed the assessment under the stringent/lenient criteria. Tertile 1 contains the lowest scoring schools

## Discussion

This paper explores the use of “common content” items shared across UK medical schools, embedded in the knowledge test components of high-stakes, graduating level assessment. We show that candidates from different medical schools exhibit significant differences in scores on common content, and that these differences are partly generalisable – with schools differing across many domains. Importantly, a like-for-like comparison shows scores vary by enough that standard setting approaches that produce realistic fail rates – that is, fail rates that match those reported in similar assessments and for medical schools [29, 30] – may produce substantially different fail rates despite identical content and candidates being governed by the same regulatory environment. It is important for all medical educators – including those responsible for clinical teaching – to be aware of such trends and to contribute to ongoing discussions on how to reach a consensus on standard setting for national licensing assessment. Even if the standards here are taken as illustrative only, the observed variation in hypothetical passing rates emphasises the need for medical educators to agree whether standards should be uniformly applied, or locally-determined – as either approach will have substantial practical implications for any cross-institutional assessment. Within this discussion it will be important to reach a consensus on the minimally acceptable standard among all stakeholders – and determine whether the current approaches to training new doctors [19, 20] will be assisted by a licensing assessment. However, it is possible that the observed variation here reflects genuine differences in performance by schools in the top and bottom tertiles. If so, this could support the argument for the application of a national ‘minimally acceptable’ standard, albeit with complex consequences for schools at the extremes of performance, as the paper will next explore.

These findings extend and support previous research. They suggest that differences found in postgraduate attainment [6] may be partly attributed to differences in undergraduate medical education or attainment. The limited previous evidence of attainment variation on common content has been reinforced [13]. The emerging consensus that standard setting is a highly localised and subjective process influenced by contextual factors including local curricular differences [11, 12] offers insights into the attainment differences found here. Schools may be emphasising different areas and levels of knowledge, which then leads to significant differences on a shared assessment.

The evidence suggests that a common set of passing standards would impose high (or low) pass rates on some schools. That this is not happening currently could be explained by standard setters being heavily influenced by the performance of their local students rather than applying an arguably more objective national standard. Alternatively, it could be that material outside the common content is unique – implying less equivalence across schools. Differences in common content scores between schools may be due to differences in cohort ability, or variations in the format and emphasis of assessment at each institution.

If medical schools have divergent standards due to “localisation,” significant disruption may occur if a single national standard is imposed. This may have substantial effects on passing rates and may disrupt workforce supply or affect stakeholder confidence in the exit assessment unless all stakeholders can work together to develop a sufficiently flexible approach that is acceptable to everyone.

This work shows that a shared regulatory environment alone does not necessarily develop homogeneity of performance, though it may have set an effective minimum standard if the standards of the lowest-performing medical school were found to be acceptable to all stakeholders. Importantly, however, given the known passing rates of UK medical schools, were such a “minimum standard” acceptable it would raise the concern that high-performing medical schools may be failing candidates who would be considered of passing quality by that minimum standard.

The extent to which educational diversity in content knowledge and topic specialisation is a desirable outcome [17] or a problem requiring regulation needs further discussion among educators and stakeholders. Either way, the experience of national assessment elsewhere suggests inevitable disruption during the implementation period [21–24].

The underlying ambiguity around current standard setting processes emphasises a challenge to medical education itself. If ongoing research on standard setting and empirical evidence suggests standard setting is not reproducible across time and contexts [11, 12] we must consider the impact on defensibility of assessments. We cannot judge from this work whether highly scoring medical schools are too stringent or whether lower scoring medical schools are too lenient or whether they are simply different in ways current regulatory processes fail to identify. It is extremely difficult to establish if there is a “correct” approach in a complex environment, and involvement of stakeholders throughout institutions affected by national licensing assessment is necessary.

## Strengths and limitations

This study has several methodological strengths. The items have been reviewed and audited by experts then sat by many candidates across many institutions. This led to a high-quality dataset covering almost all candidates within a single regulatory environment. Our ability to compare schools on shared subsets of items allowed for a rigorous estimation of the impact of different standard setting regimes using empirical data. As such it serves as a plausible model for a future licensing assessment. The developers of the common content project (MSCAA) have a significant role in developing the national licensing assessment for UK medical schools, and items similar to those selected in this study are likely to be used in the licensing assessment itself, adding further rigour to the work described. Importantly, we have opted for a widely understood, simple analytical approach via Classical Test Theory to make the results accessible to the largest possible audience of medical educators, policy makers and other stakeholders.

Despite this, there were limitations. The pool of items is smaller than would be expected in a full-sized examination, and candidates also sat locally developed items which could not be included in this analysis. Some schools used relatively few common content items and the mechanism by which schools select or reject items – or how they are integrated into wider assessment and teaching – remains underexplored. This study uses common content instead of a licensing assessment, and so a complete licensing assessment might exhibit different patterns of results. The comparisons made in part 1, while useful, were based on different items representing different content domains and may have varied in difficulty level. The study did not incorporate admissions data, and so cannot determine the extent to which cohort differences in early academic performance explain the variance in common content scores. Finally, while the accessibility of the work is a positive, more advanced methods such as Rasch inevitably offer additional analytic tools not employed in this analysis [27]. However, it should be noted that the Rasch model of this dataset did not contradict any of the findings set out here.

### Future research

Future research should explore the stability of these trends and expand the availability of common content material to better compare medical schools. It is important to identify the mechanisms behind these differences (for which controlling for admissions or other assessment scores is especially important), and to ensure that a broad range of medical schools across the spectrum of performance are involved in standard setting any proposed licensing assessment. More generally, the subjectivity of standard setting methods suggests we must more thoroughly explore the link between performance at medical school and performance in the workplace – to see how graduates of different ability levels perform in work. Doing so will help ensure undergraduate medical education are appropriate to the role(s) candidates are trained for.

Throughout this paper we have noted the tension between the promotion of educational diversity (often prized by medical educators) and the need to ensure rigorous minimum safety standards. As part of the development of licensing assessments it would be beneficial for researchers to consult widely with patients to ensure licensing assessments can best meet public needs. Such work could include how best to manage educational diversity and how to approach cross-institutional differences in attainment. More research on this important policy area would be very useful.

## Conclusions

This study has highlighted differences in performance across UK medical schools. It is essential all stakeholders work together to better understand these differences and determine the extent to which the differences reflect desirable educational diversity – or indicate a need for change.

## Data Availability

Due to the confidentiality and sensitivity of high-stakes assessment data, the datasets described in this study are not publicly available. If you wish for more information about the dataset or study, please contact David Hope (david.hope@ed.ac.uk).
